# Effect of Selected Commercial Plasticizers on Mechanical, Thermal, and Morphological Properties of Poly(3-hydroxybutyrate)/Poly(lactic acid)/Plasticizer Biodegradable Blends for Three-Dimensional (3D) Print

**DOI:** 10.3390/ma11101893

**Published:** 2018-10-03

**Authors:** Přemysl Menčík, Radek Přikryl, Ivana Stehnová, Veronika Melčová, Soňa Kontárová, Silvestr Figalla, Pavol Alexy, Ján Bočkaj

**Affiliations:** 1 Institute of Material Chemistry, Faculty of Chemistry, Brno University of Technology, Purkyňova 464/118, 612 00 Brno, Czech Republic; mencik@fch.vut.cz (P.M.); prikryl@fch.vut.cz (R.P.); xcstehnova@fch.vut.cz (I.S.); xcmelcova@fch.vut.cz (V.M.); xcfigallas@fch.vut.cz (S.F.); 2 Institute of Natural and Synthetic Polymers, Faculty of Chemical and Food Technology, Slovak University of Technology in Bratislava, Radlinského 9, 812 37 Bratislava, Slovakia; pavol.alexy@stuba.sk (P.A.); jano.bockaj@gmail.com (J.B.)

**Keywords:** plasticizers, poly(hydroxybutyrate)/poly(lactic), mechanical properties, thermal properties, biodegradable polymeric blends, 3D printing

## Abstract

This paper explores the influence of selected commercial plasticizers structure, which are based on esters of citric acid, on mechanical and thermal properties of Poly(3-hydroxybutyrate)/Poly(lactic acid)/Plasticizer biodegradable blends. These plasticizers were first tested with respect to their miscibility with Poly(3-hydroxybutyrate)/Poly(lactic acid) (PHB/PLA) blends using a kneading machine. PHB/PLA/plasticizer blends in the weight ratio (wt %) of 60/25/15 were then prepared by single screw and corotating meshing twin screw extruders in the form of filament for further three-dimensional (3D) printing. Mechanical, thermal properties, and shape stability (warping effect) of 3D printed products can be improved just by the addition of appropriate plasticizer to polymeric blend. The goal was to create new types of eco-friendly PHB/PLA/plasticizers blends and to highly improve the poor mechanical properties of neat PHB/PLA blends (with majority of PHB) by adding appropriate plasticizer. Mechanical properties of plasticized blends were then determined by the tensile test of 3D printed test samples (dogbones), as well as filaments. Measured elongation at break rapidly enhanced from 21% for neat non-plasticized PHB/PLA blends (reference) to 328% for best plasticized blends in the form of filament, and from 5% (reference) to 187% for plasticized blends in the form of printed dogbones. The plasticizing effect on blends was confirmed by Modulated Differential Scanning Calorimetry. The study of morphology was performed by the Scanning Electron Microscopy. Significant problem of plasticized blends used to be also plasticizer migration, therefore the diffusion of plasticizers from the blends after 15 days of exposition to 110 °C in the drying oven was investigated as their measured weight loss. Almost all of the used plasticizers showed meaningful positive softening effects, but the diffusion of plasticizers at 110 °C exposition was quite extensive. The determination of the degree of disintegration of selected plasticized blend when exposed to a laboratory-scale composting environment was executed to roughly check the “biodegradability”.

## 1. Introduction

Bioplastics have gained significant attention from the researches, manufacturers, customers, and investors worldwide over the past 25 years as the bio-alternatives for various fossil-resource based products. The pollution of the environment that is caused by plastics, the disappearance of landfill space, diminishes of petroleum resources, and the interest in controlling the toxic gases emissions are ranked among the reasons for this attention. The term “biopolymer” refers to polymers that are bio-based, biodegradable, or both; so, among the benefits of bioplastics from biopolymers that are based on renewable resources (or even on waste materials) is reduced carbon dioxide production during the bioplastics synthesis and their biodegradability [1,2,3,4].

Biobased material has some or all of its carbon produced from renewable resources like corn or sugar cane, or is produced in yeast, bacteria, or algae. The conditions for the determination of biobased content of polymeric materials are described in the European standard CEN/TS 16295:2012 [4].

Under appropriate conditions of moisture, temperature, and oxygen availability, the biodegradation leads to the fragmentation or disintegration of plastics with no toxic or environmentally harmful residue [5]. According to CEN/TR 15932:2010, biodegradable polymers are able to completely degrade to low molecular products (carbon dioxide, methane, water) and the biomass (mineralization and bioassimilation) through the chain scission in the backbone [6]. Specific test conditions, environments, and time-scales for laboratory tests are defined in European standards (EN ISO 14851:2004, EN ISO 14852:2004, EN ISO 17556:2004, EN ISO 14855-1:2007/AC:2009, EN ISO 14855-2:2009) [7,8,9,10,11]. Complete biodegradation is a combination of physical and enzymatic processes (where the chain scission is caused by human, animal, or fungi cell activity). The biodegradation progress is monitored by the measurement of oxygen uptake and carbon dioxide production. A special case of biodegradability is the compostability, where the degradation of a sample takes place in a soil being strongly influenced by composting conditions, such as the substrate properties, the microbial activity, the water content, and pH (EN ISO 13432:200/AC:2005, EN ISO 14995:2006) [4,12,13].

Although high costs of biodegradable polymers and the lack of regulation and composting facilities still prevent the massive distribution of bioplastics, the hypothetical global bioplastics production capacity is expected to increase from around 2.05 million tons in 2017 to approximately 2.44 million tons in 2022 [1,14]. Generally, bioplastics are used in a growing number of markets, from packaging, catering products, consumer electronics, automotive, agriculture/horticulture, toys and textiles to medical applications, and a number of other segments. Well-investigated biopolymers, such as PLA and PHAs, are the main drivers of production capacity growth [14].

PHA includes a group of microbial polyester polymers and copolymers (150 different PHA monomers have been reported yet). These bio-based biodegradable linear polyesters feature a wide array of physical and mechanical properties depending on their chemical composition. The occurrence of short-chain-length PHA (scl-PHA) with 3–5 carbon atoms per unit is the most common [15,16]. The PHAs production is estimated to triplicate the capacities in next five years [14]. PHAs are stored as intracellular carbon and energy reserve granules in diverse Gram-negative and Gram-positive bacteria from a range of substrates, including sugars and fatty acids [15].

Poly(lactic acid) (PLA), –[CH(CH_3_)COO]_n_– is very versatile aliphatic polyester that features excellent barrier properties and is available in the form of high-performance PLA grades that are a good replacement for PS (polystyrene), PP (polypropylene), and ABS (acrylonitrile butadiene styrene) in more demanding applications [14,17]. PLA is synthesized from renewable resources through lactic acid fermentation and it is highly transparent, colorless, glossy, and rigid thermoplastic material [18,19]. The properties of PLA are dependent on the ratio between D and L enantiomers. The disadvantages of this polymer are its low heat distortion temperature (softening above 60 °C) and also its inability to quickly biodegrade at ambient temperature [18]. The tensile properties of PLA can vary widely depending on whether it is annealed or oriented, or on its degree of crystallinity [19,20]. 

The combination of these non-toxic, biodegradable, and biocompatible materials (PHAs, PLA) with three-dimensional (3D) printing technologies is useful, especially for the biomedical field (scaffolds, implants), art objects, precise replicas of archeological objects, and for prototyping and the manufacture of spare parts for automotive, airplanes, etc. [17]. PLA, as biodegradable polymer, is already used in 3D printing industry. Biodegradable PLA/PHA polymeric blends are also applicable for 3D printing, even in industry at the time [21]. Their printability is good.

Fused Deposition Modeling (FDM) is the most productive technology used in low-cost 3D printers. A plastic filament used in this method is pushed through heated extrusion nozzle, melted and deposited on the bed in a tailored shape. The software that processes a STL (stereolithography) or CAD (computer-aided design) file, mathematically slicing and orienting the model for the building process, is embodied in this method [22]. The FDM method is applied in this work for the preparation of 3D printed dogbones (double-paddle shaped testing elements), which are further used for tensile tests. Usually, it is necessary to deal with the warping problem, which occurs especially during the printing of large parts, where the internal temperature of printed object tends to vary. Our materials are printed at high temperature (190 °C, the temperature of the extrusion nozzle) onto the printing bed (which has ambient temperature) and cooled with an integrated fan for quick solidification. This usually causes the shrinkage and it can lead to the deformation through the warping, which can lead to the detachment from printing bed and to the product destruction [23]. The warping wasn´t relevant in the case of our 3D printed dogbones.

Although PLA and Poly(3-hydroxybutyrate (PHB) can be potentially used as biodegradable and biocompatible alternatives to conventional polymers by many industrial sectors, both PLA and PHB have a few disadvantages. PHB is tough, fragile, and brittle in nature and it has poor processing properties. Blending with amorphous polymer, such as poly(d,l-lactic acid), is proposed to reduce the crystallinity of PHB, so that the applications of PHB can be expanded [3,24]. In spite of blending, these neat PHB/PLA blends are still stiff and brittle materials with poor mechanical properties and thermal degradation close to its melting point, limiting its processability [25]. Poor ductile properties can be well improved by the addition of plasticizer.

Plasticizers are an important class of low molecular weight non-volatile compounds that were homogeneously incorporated in a material (usually plastics or elastomer) that are widely used in polymer industries as additives. The primary role of such substances is lowering the glass transition temperature (*T*_g_), the melting temperature (*T*_m_), and improving the flexibility, the distensibility, and the processability of biopolymers [26,27]. Plasticizers reduce the tension of deformation, hardness, density, viscosity, and electrostatic charge of a polymer, and at the same time, they increase the polymer chain flexibility, resistance to fracture, and dielectric constant [26,28]. The additions of plasticizer thus influence the mechanical properties—reduce the toughness, the tensile strength, and increase the elongation at break. Other properties are also affected, such as the degree of crystallinity, optical clarity, electric conductivity, fire behavior, and the resistance to biological degradation, amongst other physical properties [28]. Among the most significant plasticizers are various monomeric esters of phthalic, phosphoric, adipic, sebacic, citric, and ricinoleic acids, furthermore chlorinated paraffins and low-molecular weight polyesters [26,27].

Plasticizers can be divided into two principal groups: primary and secondary. Primary plasticizers lower the glass transition temperature *T*_g_ and increase the elongation and softness of the polymer. Secondary plasticizers, when added to the polymer alone, do not bring about such changes and may also have limited compatibility with the polymer. However, when added to the polymer in the presence of a primary plasticizer, secondary plasticizers enhance the plasticizing performance of the primary plasticizer [29]. In this current study, the primary plasticizers that externally plasticized the polymeric blends were used.

The principle disadvantage of external plasticizing is the migration of the plasticizer from the plasticized material to other contacting media. This process includes the diffusion of the plasticizer from bulk material towards the surface, the interface phenomena, and the sorption or evaporation into the surrounding medium [28]. One of the consequences of the plasticizer decrement is the loss of the material elasticity and ductility. In addition, the plasticizer leakage from the material can be the cause of surrounded media contamination (medical application issues). Among the main factors that affect the plasticizer migration are the type and the concentration of the plasticizer, its molecular weight, branching, and polarity. Low-molecular weight plasticizers tend to leak away from the polymeric material easier. The more linear the plasticizer’s structure, the more extraction rate and migration occurred when compared to more branched plasticizers. The plasticizer migration is also affected by the type of polymer, its molecular weight, and its compatibility with the plasticizer; and, by the plasticization process and homogeneity of the product. The migration is also influenced by the conditions of the migration test (type of contact, time, temperature, polymer probe, specimen thickness). Higher temperatures simplify the plasticizer migration, while lower temperatures decelerate the migration [27,29,30,31,32,33,34,35].

In this current study, four commercial monomeric plasticizers that are based on esters of citric acid were used. The plasticizers miscibility with PHB/PLA blends in the weight ratio (wt *%*) of 85 (polymer)/15 (plasticizer) was confirmed using a kneading machine. The PHB/PLA/plasticizer blends in the weight ratio of 60/25/15 were then prepared by single screw and corotating meshing twin screw extruders in the form of filaments (for further 3D printing). The mechanical properties of plasticized blends were determined by tensile test of filaments as well as 3D printed dogbones. The diffusion of plasticizers from blends that were exposed at 110 °C in the drying oven was also investigated. The determination of the degree of disintegration of selected plasticized blend when exposed to a laboratory-scale composting environment was executed to roughly check their biodegradability. The Modulated Differential Scanning Calorimetry (MDSC) was performed to check the thermal properties of the plasticized blends and Scanning Electron Microscopy (SEM) was used to check their morphology. The goal was to create new types of eco-friendly PHB/PLA/plasticizers blends and to highly improve poor mechanical properties of neat PHB/PLA blends (with majority of PHB), by adding appropriate plasticizer. 

These environmentally friendly PHB/PLA/plasticizer blends with the majority of PHB are biocompatible and suitable for 3D printing technology, and thus for biomedical applications and prototyping. Additionally, such type of eco-friendly blends, containing the PHB majority, can be well compostable. PHB can be prepared, even from waste cooking oil, so, unlike other types of biodegradable bioplastics, it does not require the agricultural feedstock, such as corn, potato, sugar cane, or sugar beet [36,37]. Quite many plasticizers were tested with PHB or PLA apart to improve their thermal and mechanical properties for various applications. Only some studies have focused on improving the thermal and mechanical properties of PHB/PLA/plasticizer blends, but always with the PLA majority. 

Armentato et al. [38] investigated binary and ternary films made of PLA, 15% PHB and OLA (oligomeric lactic acid), which were prepared under optimized extrusion conditions, followed by a filmature procedure. The mechanical, thermal, morphological, and functional properties were investigated as the function of OLA composition ratio (15, 20, and 30 wt % of OLA by weight).

Arrieta et al. [25] blended PLA with PHB (75:25 wt % ratio) to increase the crystallinity. The plasticizers acetyl(tributyl citrate) (ATBC) and poly(ethylene glycol) (PEG) were added in the amount of 15 wt % when PLA/PHB blends achieved the melt state, to increase the processability and ductility. Each blend was then processed into biodegradable flexible film with improved properties for food packaging.

Abdelwahab et al. [3] investigated a blend of PLA (75% by weight) and PHB (25%) with a polyester plasticizer (Lapol 108) at two different concentrations (5 and 7% by weight per 100 parts of the blends).

This paper describes the influence of four plasticizers on thermal, mechanical, and morphological properties of plasticized PHB/PLA blends, but with the PHB majority. This kind of work has not been likely published yet, and thus it is unique in this context.

## 2. Materials and Methods

### 2.1. Materials

Poly-3-hydroxybutyrate (P3HB Biomer^®^, *ρ* = 1.23 g∙cm^−3^, *M*_w_ = 410,000 g·mol^−1^) was provided by Biomer Company (Krailling, Germany) [39]. The polymer was in the form of white powder.

The PLA used in this study is poly(d,l-lactic acid), which is amorphous at *T_g_* around 60 °C. This amorphous PLA was chosen for its ability to reduce the overall crystallinity content in our PHB/PLA/plasticizers blends, which are further used for 3D printing. Amorphous polylactic acid (aPLA) granules (Ingeo™ 4060D, *M*_w_ = 180 000 g·mol^−1^, *T*_g_ = 55–60 °C, *ρ* = 1.24 g∙cm^−3^) were supplied by NatureWorks LLC (Minnetonka, MIN, USA) Company [40].

**Figure 1:**
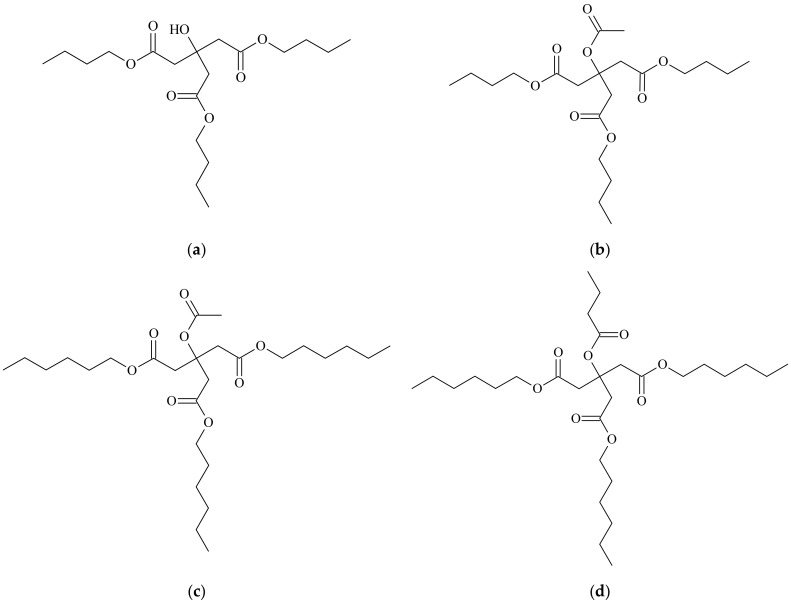
(**a**) Plasticizer C-4 structure; (**b**) Plasticizer A-4 structure; (**c**) Plasticizer A-6 structure; and (**d**) Plasticizer B-6 structure.

Four monomeric commercial plasticizers that are selected for this study are based on esters of citric acid, as provided by Vertellus Holdings LLC Company (Indianapolis, IN, USA), with trademark Citroflex^®^ [41]. Their commercial and chemical names, molecular weights, and labels can be seen in Table 1. Their structure can be seen in Figure 1. 

### 2.2. Plasticizers Miscibility with PLA/PHB Polymers

The miscibility of plasticizers with PHB/PLA polymer mixture was tested using the kneading machine PLASTI-CORDER Brabender. Citroflex^®^ plasticizers were kneaded with PHB/PLA mixture in the weight ratio (wt %) of 60 PHB:25 PLA:15 plasticizer. Non-plasticized PHB/PLA was kneaded as a reference for the comparison (70 PHB:30 PLA). The total weight of each sample before the kneading process was 30 g. The process of kneading plasticizers with the PHB/PLA mixtures was performed at 178–180 °C (chamber temperature) at 45 rev./min, and the kneading process of reference was performed at 180 °C. The PHB/PLA mixture was dosed into the PLASTI-CORDER Brabender machine (Brabender GmbH & Co. KG, Duisburg, Germany) first and then kneaded for 1.5 min. Then, the plasticizer was added and the kneading process continued for following 3.5 min in the case of C-4, A-4 and B-6 plasticizers and for 4.5 min in the case of A-6 plasticizer. The total kneading time of PHB/PLA with plasticizers (sample dosing with kneading) was 6–7 min. The total kneading time of reference was 9 min. The gearbox torque dependence on the total plasticization time was measured and the miscibility was evaluated.

### 2.3. Preparation of Plasticized PHB/PLA Blends

PHB and PLA polymers were firstly pre-treated by drying at 80 °C in the drying oven (Memmert, Schwabach, Germany) for 2 h to eliminate possible absorbed water. As the first step, the corotating meshing twin screw extruder with the screw auger parameters D = 16 mm and L/D = 40, from Labtech Engineering Company (Samutprakarn, Thailand), was used for the preparation of plasticized PHB/PLA blends. The total weight of each sample for extrusion was 350 g. The chamber temperature profile from hopper to nozzle was set to 160–175–175–75–175–175–175–170–165–160 °C and the rotational speed of screw was set to 130 rev./min. The PHB/PLA blends in the weight ratio of 70/30 (wt %) (reference) as well as the PHB/PLA/plasticizer blends in the weight ratio of 60/25/15 (wt %) were extruded in the form of filaments. Four Citroflex^®^ plasticizers were used one by one. Unfortunately, the filament, which was pulled from the nozzle, had diverse non-defined thickness and diameter and so it was unsuitable for 3D printing. Therefore, the filaments were pulled through the water tank (water with ambient temperature) and then the filaments were pulled into the granulator to get pellets, which are later used for the second extrusion.

The PHB/PLA (reference) pellets and the PHB/PLA/plasticizer pellets that were prepared by twin screw extruder, were at first pre-treated by drying at 80 °C for 2 h in the drying oven. Then single screw extruder HAAKE^TM^ Rheomex OS (Haake Technik GmbH, Vreden, Germany) was used for the second extrusion in order to achieve the filaments with a defined diameter of 1.75 mm. The chamber temperature profile from hopper to nozzle was set to 185–175–170–150 °C and the rotational speed of screw was set to 25 rev./min. The filament that extruded from the nozzle was pulled out into the draw-off device through the calibration mechanism located in the water tank. Water was tempered by the thermostat at 60 °C in order to finish the polymer crystallization. Prepared PHB/PLA/plasticizer filaments with defined diameter were then used for further characterizations and for 3D printing.

### 2.4. Modulated Differential Scanning Calorimetry—Thermal Characterization

The thermal properties of prepared samples were determined using the combination of modulated and conventional differential scanning calorimetry (MDSC and DSC, respectively). The measurements were performed on TA Instruments model DSC 2500 (TA Instruments, New Castle, DE, USA) under nitrogen atmosphere. The samples (approx. 13 mg) were sealed in hermetic aluminium pans. Firstly, they were cooled down to −50 °C and then kept in isothermal mode for 5 min. Afterwards, MDSC scan from −50 to 80 °C was carried out at average heating rate of 3 °C/min with the period of 60 s and modulation amplitude of 1.25 °C. This ensured that measured properties corresponded to as prepared samples without further thermal stress. Subsequently, two DSC scans (heating rate 10 °C/min), the first from 80 to 190 °C and the second from −30 to 190 °C, were performed. The glass transition temperature *T*_g_ was determined from the MDSC curve of reversing heat capacity. The melting temperature *T*_m_ and the crystallization temperature *T*_c_ were determined from the conventional DSC scans. The crystallinity *X*_c_ was calculated from the measured data using the following equation:

(1) $$X_{c}=\frac{\Delta H_{m}}{\Delta H_{m}^{0}}\cdot 100\%,$$

where Δ*H_m_*, and $\Delta H_{m}^{0}$ (J/g) are the enthalpy of second melting peak and the enthalpy of fusion of 100% crystalline polymer (146 J/g for PHB), respectively.

### 2.5. Migration Tests—Plasticizers Diffusion from PHB/PLA Blends

First experiments with the plasticizers diffusion from our blends were performed by the Thermogravimetric Analysis (TA Q500, TA Instruments, New Castle, Delaware USA). However, it was not possible to determine the diffusion speed exactly, as some of the plasticizers showed too long evaporation from the surface. Therefore another test was conducted. 5 g of extruded PHB/PLA/plasticizer pellets and 5 g of extruded PHB/PLA pellets (reference) were compression moulded on a laboratory hotpress to obtain planar samples of 0.70–0.72 mm thickness. The heating temperature of press desk was set to 190 °C. The laboratory press with the sample was closed during 0.5 min and the presswork itself lasted for 1 min. The samples of 3 × 3 cm square shape proportions were then cut from these moulded pressed planar samples, wiped by the napkin paper moistened in ethanol, laid down on the PET foil, and put into the drying oven heated to 110 °C with 50% air circulation. The samples were taken out from the drying oven after 1, 3, 7, 10 h, and 1, 2, 3, 6, 8, 10, 13, 15 days, and then they were wiped by the napkin paper moistened in ethanol and measured while using analytical balance. The dependencies of plasticizer’s weight loss from PHB/PLA planar samples on the time of exposition to 110 °C were evaluated.

### 2.6. Tensile Tests—Mechanical Characterization

The elongation at break, the Young´s modulus, and the tensile strength were determined under ambient conditions, using the universal measurement device Zwick Z 010 (ZwickRoell GmbH & Co., Ulm, Germany). The tensile tests of non-plasticized extruded PHB/PLA samples (references), as well as plasticized PHB/PLA samples, were carried out using the load indicator with the maximal tensile force of 500 N and with pneumatic grips. The deformation rate was initially set to 5 mm/min to determine the elastic modulus, and then enhanced to 50 mm/min. The measurements were carried out without using the extensometer. The filaments with defined diameter were tested after three days of exposition to 110 °C in the drying oven and after 7 and 40 days after their preparation by extrusion. Double-paddle testing samples (dogbones, ~(5 × 2 × 75) mm, tensile test measurements approach CSN EN ISO 527-1) were 3D printed from mentioned extruded filaments (PHB/PLA reference sample and all PHB/PLA/plasticizer samples), and they were measured using Zwick Z 010 after seven days following the 3D printing [42]. The reported values were the average of at least seven measurements. The data points were the mean value of each measurement, with the error bars in each graph representing the standard deviation.

### 2.7. 3D Printing

The dogbone samples for tensile tests were printed on PRUSA i3 MK2 3D printer (Prusa Research s.r.o., Praha, Czech Republic) by the FDM technology from PHB/PLA/plasticizer filaments with defined diameter, as mentioned above. AutoCAD (ver. 2018, Autodesk Inc., San Rafael, CA, USA) and Slic3r (software version 1.3.0, free software, developed by Alessandro Ranellucci) program were used for 3D virtual modeling and mathematical slicing. The dogbones were printed at 190 °C (195 °C during the first layers printing for better adhesion to the printing bed), at ambient conditions, without additional air cooling and bed heating. 3D printing was executed approximately six months after the filaments preparation.

**Figure 2:**
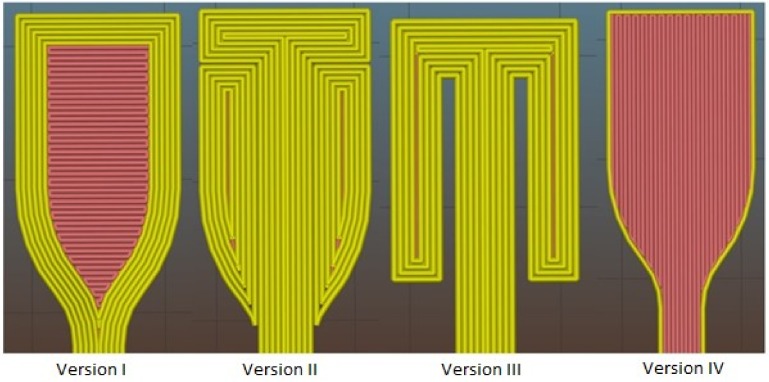
Evolution of three-dimensional (3D) printed dogbones prepared for tensile tests.

The evolution of 3D printed dogbone models that were prepared for further tensile tests can be seen in Figure 2.

The Version I was created by simple import of fabricated model to Slic3r program and uploading to the printer. But, during the tensile test with dogbones of Version I, the delamination of perimeters occurred (illustrated by yellow color), and so the measured samples did not provide relevant results. To solve this problem, Version II was designed, where the perimeters of dogbone neck were elongated up to the upper part. The paddleboards were created from two parts. Unfortunately, during the tensile test, the defect at the point of paddleboard and neck connection occurred, because the printer created an inclusion in the paddleboard corner by releasing a large amount of melting. The inclusion acted as a disruption initiator and cracked untimely. The aim of Version III was to eliminate this problem, but the pressure of grips was not strong enough to hold the sample. The deformation then occurred in upper cross-sectional part and disagreed with real dogbone strength. The final Version (that could not be illustrated in Figure 2) was created by the combination of 10 laminas of Version I and IV, which alternate regularly (five from each Version). The delamination of the neck in its extending part was eliminated by the insertion of vertical infill (illustrated by red color) and the vertical fracture of samples was eliminated by the combination with horizontal infill.

### 2.8. Scanning Electron Microscopy—Morphology Characterization

The Scanning Electron Microscopy (SEM) micrographs of dogbone areas that were fractured after tensile tests were obtained with the EVO^®^ LS 10 (Zeiss Company, Oberkochen, Germany), operated at 10 kV. The samples were coated with a gold-palladium layer in vacuum conditions to increase their electrical conductivity. The micrographs were registered at 100× magnification. This method was used for the following samples (in wt %): 70PHB/30PLA (reference) and 60PHB/25PLA/15plasticizer samples (where all four Citroflex^®^ plasticizers A-4, C-4, A-6, B-6 were used).

### 2.9. Determination of the Degree of Disintegration

The determination of the disintegration of plastic materials under simulated composting conditions in a laboratory-scale test was executed according to IS/ISO 20200 [43]. Unfortunately, the method is not applicable to the determination of real biodegradability of plastic materials under garden or industrial composting conditions. The solid matrix used consists of synthetic solid waste inoculated with matured compost that was taken from a commercial garden composting plant. The pieces of plastic test material are composted with this prepared solid matrix. The degree of disintegration is determined after a composting cycle, by sieving the final matrix through a 2 mm sieve in order to recover the non-disintegrated residues. The reduction in mass of the test sample is considered as disintegrated material and is used to calculate the degree of disintegration [43]. The composition of synthetic waste used in this method was: sawdust, rabbit-feed, ripe compost, corn-starch, saccharose, cornseed oil, and urea. The box with a lid made of polypropylene, having the dimensions of (30 × 20 × 10) cm, was used as a composting reactor. Two defined holes were made in the middle of two 20 cm sides to provide the gas exchange between inner atmosphere and outer environment. As samples, PHB/PLA/A-4 (60/25/15 in wt %) were used in the form of thin films with the dimensions approximately (6.5 × 10 × 0.08) cm. Two identical films were located in each box. The solid matrix was prepared manually by mixing different components that are mentioned above. Each box was filled by solid matrix up to the middle. Beforehand weighed and measured samples were laid on this matrix and covered with further matrix. Prepared content was then equally washed down by distilled water to overall 55% of humidity. Each reactor was closed, weighed, and placed in the air-circulation oven maintained at constant temperature of 58 °C for a minimum period of 45 days and maximum of 90 days, so that the samples were disintegrated under thermophilic conditions. The reactors were then weighed, water was added to restore the initial mass, or to restore the mass to 80% or 70% of the initial mass, the waste was stirred, and all of these actions were executed in particular days according to IS/ISO 20200. Also, other parameters, like visual appearance, odor, and color, were observed and the photos were taken.

## 3. Results and Discussion

### 3.1. Plasticizers Miscibility with PHB/PLA

Citroflex^®^ plasticizers were kneaded with PHB/PLA mixture in the weight ratio (wt %) of 60 PHB:25 PLA:15 plasticizer, using the kneading machine PLASTI-CORDER Brabender (Brabender GmbH & Co. KG, Duisburg, Germany) equipped with two counter rotating sigma blades.

**Figure 3:**
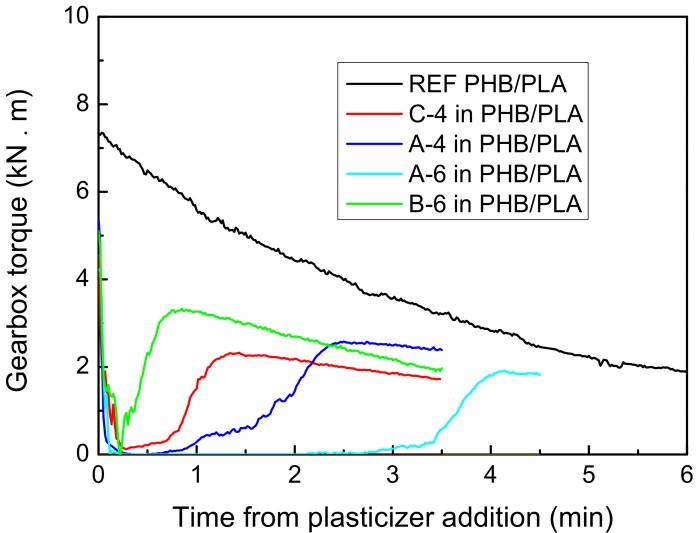
Gearbox torque dependence on the time elapsed from the addition of plasticizers to Poly(3-hydroxybutyrate/Poly(lactic acid) (PHB/PLA) mixture.

In Figure 3, you can see the gearbox torque dependence (kN∙m) on the time when plasticizer was added to PHB/PLA mixture (PHB/PLA mixture was already dosed during 1 min and kneaded for 1.5 min). These dependencies are compared with the reference sample (neat 70 PHB:30 PLA in wt %). As can be seen from Figure 3, the gearbox torque first declined to 0 kN∙m after the plasticizer addition due to the lubrication of Sigma Blades by plasticizer. The blades could move freely, and the torque resistance approached zero. Afterward, the plasticizer started to mix with PHB/PLA, so the gearbox torque increased and achieved the maximum, which corresponds to the whole mixture viscosity increase. Achieved maximum should mean the moment of plasticizer blending with PHB/PLA. Citroflex^®^ plasticizers in 15% concentration were miscible with PHB/PLA during maximum 7 min of total kneading time. It corresponds to maximum 4.5 min from plasticizer addition. All the plasticizers were successfully kneaded with PHB/PLA mixture. The plasticizers B-6, C-4 and A-4 were kneaded into PHB/PLA mixture faster than the plasticizer A-6. The influence of plasticizer structure on measured dependencies can be seen especially when the plasticizers A-4 and A-6 are compared. The plasticizer A-6 with longer polymeric chains (acetyl trihexyl citrate) needed a longer time to be kneaded into PHB/PLA mixture than the plasticizer A-4 with shorter polymeric chain (acetyl tributyl citrate). On the other hand, the most branched plasticizer B-6 (butyryl trihexyl citrate), which should theoretically be kneaded for the longest time with PHB/PLA mixture, actually needed the shortest time. Branched plasticizer B-6 seems to have weak cohesive forces and it thus tends to disperse well between PHB and PLA molecules at higher kneading temperature.

### 3.2. Differential Scanning Calorimetry—Thermal Behavior

**Figure 4:**
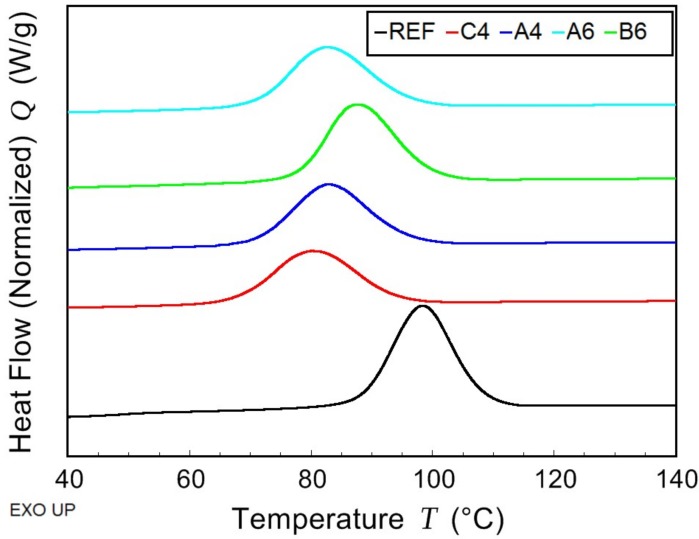
Differential scanning calorimetry (DSC)—first cooling scan of non-plasticized reference sample (REF) and PHB/PLA/Plasticizer (where the plasticizers are: C-4, A-4, B-6, and A-6).

**Figure 5:**
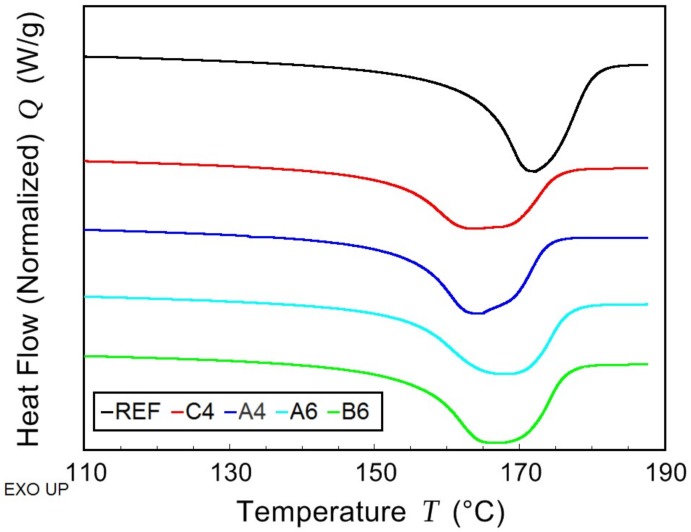
DSC—second heating scan of non-plasticized reference sample (REF) and PHB/PLA/Plasticizer (where the plasticizers are: C-4, A-4, B-6, and A-6).

**Figure 6:**
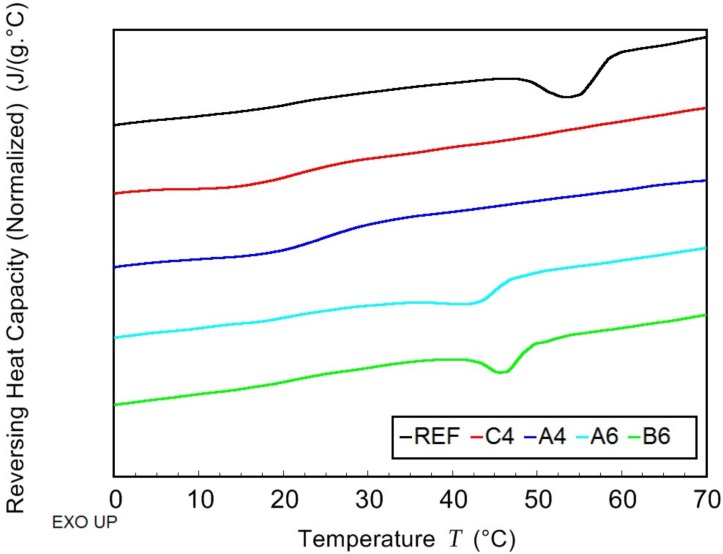
Reversing heat capacity gained from Modulated Differential Scanning Calorimetry (MDSC) scan of non-plasticized reference sample (REF) and PHB/PLA/Plasticizer (where the plasticizers are: C-4, A-4, B-6, and A-6).

The thermal behavior of PHB/PLA/plasticizer samples and non-plasticized reference sample was observed while using the combination of modulated and conventional differential scanning calorimetry (MDSC and DSC, respectively). Due to high amount of rigid fraction in PHB, its glass transition temperature could not be detected. The effect of the plasticizer on PHB thermal properties was observed by means of melting and crystallization temperatures shifts (Figure 4 and Figure 5, respectively), as well as by the crystallinity change. In the case of PLA fraction in blends, the change in *T*_g_ was monitored and it is presented in Figure 6. All the evaluated data are listed in Table 2.

The introduction of low molecular plasticizer into PHB/PLA blend caused greater mobility of macromolecular chains. As a consequence, the crystallization and the melting temperatures were shifted to lower temperatures. The most significant drop was observed in the case of C-4 (tributyl citrate), where, as compared to the reference, *T*_c_ was lower by 18 °C and *T*_m_ was lower by 9 °C. The crystallinity was reduced in all samples except for that with B-6 (butyryl trihexyl citrate). In a semicrystalline polymer, low molecular additives are retained in amorphous phase in between crystallites. In a blend of semicrystalline (PHB) and amorphous (PLA) polymer, such additives are expected to be ejected into amorphous polymer during the crystallization. Therefore, the plasticizing effect is more apparent when observing the change of PLA *T*_g_. Butyl esters of citric acid caused a decrease of PLA *T*_g_ by 35 °C for C-4 and by 33 °C for A-4 (acetyl tributyl citrate). Increased length of alkyl chains that are linked to citric acid conversely showed a worse plasticizing effect and led to *T*_g_ higher than 40 °C. At the same time, there is the effect of alkyl chain that is attached to –OH end of citrate molecule as butyryl trihexyl citrate exhibits the highest PLA *T*_g_ of all the samples. Both of the blends with B-6 and A-6 (acetyl trihexyl citrate) are expected to behave glassy at laboratory temperature.

### 3.3. Migration Tests—Plasticizers Diffusion from PHB/PLA Blends

The plasticizers diffusion from PHB/PLA/plasticizer (60/25/15 in wt %) samples was evaluated as their weight loss (%) after up to 15 days of exposition to 110 °C in the drying oven.

**Figure 7:**
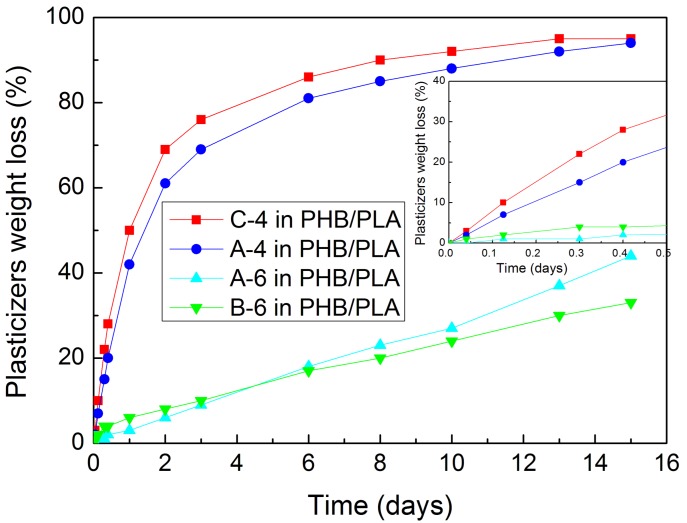
Plasticizers weight loss (%) from PHB/PLA/plasticizer blends after up to 15 days of exposition to 110 °C in the drying oven.

The weight loss (%) of C-4, A-4, A-6, and B-6 plasticizers after 1, 3, 7, 10 h, and 1, 2, 3, 6, 8, 10, 13, and 15 days of exposition to 110 °C can be seen in Figure 7. The plasticizers began to migrate rapidly after 10 h of exposition, especially in the case of C-4 (tributyl citrate) and A-4 (acetyl tributyl citrate). On the other hand, 15 days of exposition to 110 °C must be considered as a hard thermal condition for bioplastics. They are not expected to be exposed to so severe conditions during their ordinary use. The plasticizers most resistant to diffusion are B-6 (butyryl trihexyl citrate) and A-6 (acetyl trihexyl citrate), while C-4 and A-4 plasticizers had up to 95% weight loss after 15 days of exposition. Stable plasticizers B-6 and A-6 showed only 33 and 44% weight loss after 15 days of exposition and their weight loss proceeded gradually. The influence of plasticizer structure on the diffusion is well noticeable. C-4 plasticizer with –OH group, the smallest carbon amount and the least branched chain showed the fastest diffusion from the PHB/PLA/plasticizer sample. A-4, A-6, and B-6 plasticizers vary by the length of alkyl chains linked to citrate molecule and the most branched plasticizer B-6 with the largest molecular weight showed the best stability and tendency not to diffuse from the sample. This influence of structure on the diffusion agrees with [28,29,30,31,32].

### 3.4. Tensile Tests—Mechanical Behavior

**Figure 8:**
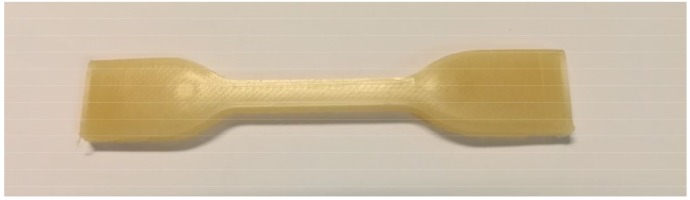
3D printed dogbone for tensile tests (PHB/PLA/A-4).

The tensile tests were measured on samples containing PHB/PLA/plasticizer (60/25/15 in wt %). Measured samples were in the form of extruded filaments and dogbones 3D printed from these filaments. The appearance of 3D printed dogbone can be seen in Figure 8.

**Figure 9:**
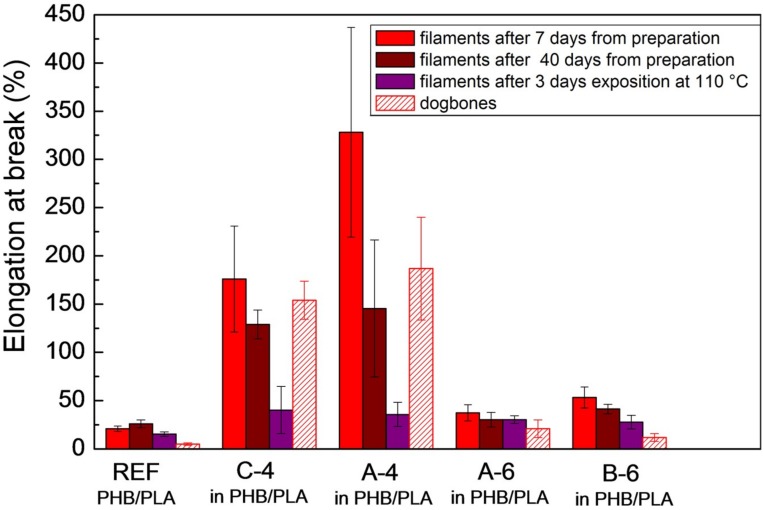
Elongation at break (%) of neat PHB/PLA (reference), PHB/PLA/plasticizers filaments, and 3D printed dogbones.

The tensile tests were carried out on filaments after seven and 40 days from extrusion, on filaments after three days of exposition to 110 °C in the drying oven and on 3D printed dogbones after seven days from printing. The results were compared with neat PHB/PLA (70/30 in wt %) as reference. With respect to plasticizers efficiency, the elongation at break is the most important value for us. In Figure 9, we can see the plasticizers influence on measured elongation at break (%) when compared with non-plasticized reference. All four Citroflex^®^ plasticizers have positive softening effect in PHB/PLA polymers.

The plasticizers A-6 (acetyl trihexyl citrate) and B-6 (butyryl trihexyl citrate) improved the elongation at break (measured after seven days from filament preparation) only slightly: from 21% for non-plasticized PHB/PLA filament samples to 37% and 53% for plasticized ones, respectively. Their plasticizing effect is insignificant, on the other hand these plasticizers are more resistant to diffusion (as was discussed in the Migration tests chapter). The results from the migration tests were also confirmed by the tensile tests measurements, since the elongation at break of blends with A-6 and B-6 plasticizers only slightly decreased after three days of exposition to 110 °C. The blends with A-6 and B-6 plasticizers are relatively resistant to the aging, as was confirmed by the tensile tests measurement after 40 days from the preparation. Weaker softening effect of these two plasticizers, but their stronger resistivity against aging and exposition to higher temperature is caused by their structure: increased length of alkyl chains linked to the citrate molecule and more branched structure. It corresponds well with the MDSC results, where A-6 and B-6 plasticizers showed minor *T*_g_ reduction, and hence worse plasticizing effect. The elongation at break of dogbones with A-6 and B-6 plasticizers confirmed a weaker plasticizing effect, which is probably due to 6-month old filament from which the dogbones were made.

On the contrary, significant plasticizing effect was achieved by using two plasticizers with the shortest length of alkyl chains: C-4 (tributyl citrate) and A-4 (acetyl tributyl citrate). The elongation at break measured after 7 days from preparation (filament extrusion) increased from 21% for non-plasticized reference to 176% for PHB/PLA/C-4 sample, and even to 328% for PHB/PLA/A-4 sample. The aging effect was most apparent for A-4 plasticizer, but still, A-4 seems to have the most significant plasticizing effect. It was confirmed by the tensile test measurements with dogbones samples. The A-4 plasticizer structure differs from C-4 only by the acetyl group that was attached to the citrate molecule. Significant plasticizing effect of C-4 and A-4 plasticizers was confirmed also by the MDSC measurements, where *T*_g_ of PLA fraction was strongly decreased in the case of samples with these plasticizers.

Meaningful plasticizing effect was depreciated by three days of exposition to 110 °C. Such thermal conditions caused massive plasticizer diffusion from the sample, especially in the case of low-molecular plasticizers with shortest alkyl chains. After the exposition, the elongations at break of samples with C-4 and A-4 plasticizers were comparable to samples containing A-6 and B-6 plasticizers. It agrees with the results from the migration test, where the samples with A-4 and C-4 plasticizers evinced an extensive diffusion. Still, the plasticizing effect was slightly preserved, even after hard thermal conditions.

The Young’s modulus (GPa) and the tensile strength (MPa) of measured PHB/PLA/plasticizer samples and non-plasticized reference can be seen in Table 3 and Table 4. Stronger softening effect of A-4 and C-4 plasticizers and their intense diffusion from samples after three days of exposition to higher temperature can be observed also from these results, for example from lower Young’s modulus values for the samples with C-4, A-4 plasticizers and the increase of these values after three days of exposition to 110 °C. The tensile strength values of plasticized samples were comparable for all the plasticizers; the change of values can be seen especially after three days of exposition to 110 °C in the case of A-4 and mainly C-4 plasticizers.

Armentato et al. [38] found that the addition of OLA produced a significant reduction of the *T*_g_ values of PLA/PHB blends and it confirmed the plasticizing effect of OLA resulting in the increase of molecular mobility of the polymer structure. Single *T*_g_ value was observed in all the studied formulations and no apparent phase separation was detected, confirming high compatibility between OLA and the PLA/PHB matrix. The introduction of OLA in 15 and 20 wt % did not result in a significant change in the PLA/PHB thermal stability. But, the thermal stability was decreased with 30 wt % of OLA. Considerable improvement of ductile properties of PLA/PHB/OLA blends was demonstrated. The introduction of OLA caused significant decrease of the tensile strength. When the OLA concentration increased, the elongation at break increased. The elongation at break was improved from 140% for non-plasticized PLA/15PHB blends to 370% for PLA/15PHB/30OLA blend. Also the oxygen and water vapor barrier properties were improved in the consequence of higher crystallinity of plasticized PLA/PHB blends. The PLA/PHB blend with 30 wt % OLA was selected as the optimum formulation, since it offered the best compromise between ductile and oxygen and water vapor barrier properties with no migration problems for the application as food packaging material [38].

According Arrieta et al. [25], their obtained PLA/PHB blends showed improved oxygen barrier properties and lowered water incorporation. PHB improved the interface interaction between PLA and plasticizers. Only one *T*_g_ value was observed for all formulations with plasticizers, suggesting the good miscibility between different components in the amorphous region. The addition of ATBC or PEG to the polymer matrices caused the *T*_g_ depression in all plasticized films. This reduction in *T*_g_ was higher in films plasticized with PEG. Decreased modulus, tensile strength, and increased elongation at break were caused by both plasticizers. Blends that were plasticized with ATBC showed higher flexibility and thermal stability than those with PEG (elongation at break improved from 2.0% for the PLA/PHB blend to 182% for the PLA/PHB/ATBC blends). PLA/PHB/ATBC blends showed higher oxygen barrier properties and hydrophobicity than those with PEG and appeared to be more effective [25].

Abdelwahab et al. [3] investigated the thermal and mechanical properties of their blends. The DSC curves of PLA/PHB blends exhibited two *T*_g_ values, which indicated that these blends were immiscible. However, the addition of Lapol to the system decreased the *T*_g_ values of PHB and PLA in the system and slightly increased the crystallinity and the temperature corresponding to the onset of decomposition for a polymer. It was attributed to higher thermal stability of Lapol. The Young´s modulus and the tensile strength were decreased by the addition of Lapol in PLA/PHB system, while the elongation at break only slightly increased (from 7% to 15%) [3].

### 3.5. Scanning Electron Microscopy—Morphology Characterization

**Figure 10:**
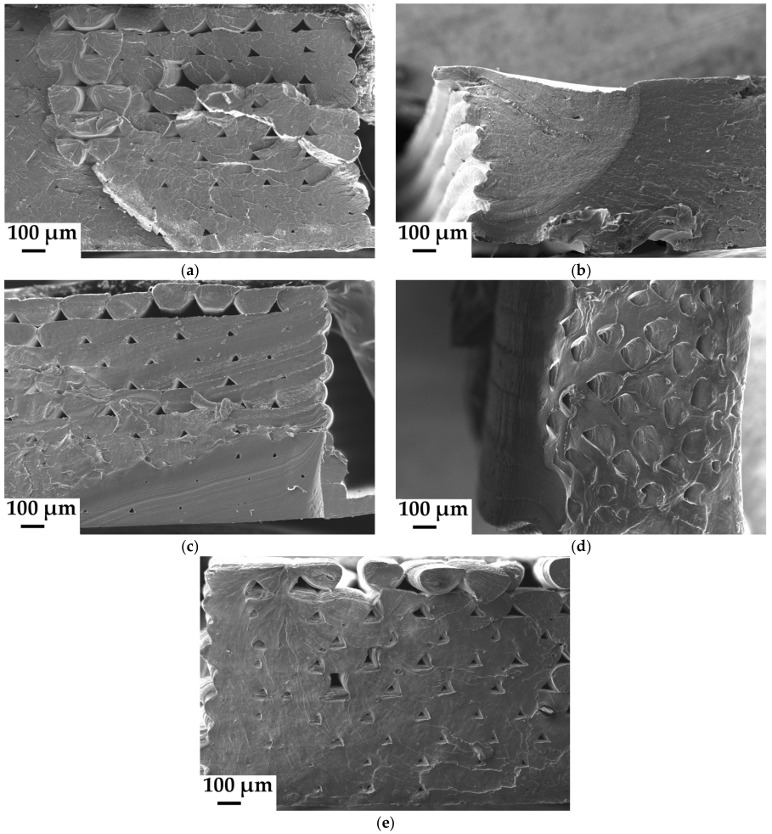
Scanning Electron Microscopy (SEM) micrographs of 3D printed dogbones fractured during tensile tests and made of: (**a**) PHB/PLA (reference); (**b**) PHB/PLA/C-4; (**c**) PHB/PLA/A-4; (**d**) PHB/PLA/A-6; and, (**e**) PHB/PLA/B-6.

The SEM micrographs of 3D printed dogbones areas that were fractured after the tensile tests can be seen in Figure 10. The micrographs were registered only at 100× magnification, but almost complete views on fractured areas were obtained. The PHB/PLA reference blend (70/30 in wt %, Figure 10a) seems to have homogeneous surface, with no apparent phase separation, suggesting the good interaction between both polymers, despite larger PHB content. However, the reference fracture surface is not very smooth; the laminas extracted during the tensile test are evident. There are also apparent holes, caused by 3D printing. The holes are larger especially near the top of the sample, where the filament laminas did not fit tightly. The laminas located near the bed and printed earlier are better fused, linked and pressed by upper laminas. This effect is apparent even from the micrographs of plasticized blends, see Figure 10b–e (60PHB/25PLA/15plasticizer in wt %). Although mechanical properties of non-plasticized (reference) and plasticized samples are different, their fracture areas on micrographs seem to be quite similar. Assumed ductile fracture patterns are not so apparent on the micrographs with plasticized samples. Micrographs seem to be similar compared to assumed rigid fracture of non-plasticized reference. The plasticizers with strong softening effect, like C-4 (tributyl citrate) and A-4 (acetyl tributyl citrate), seem to be well miscible with the polymer matrix, the surface is more compact and smooth, especially in case of C-4 plasticizer (Figure 10b,c). It corresponds well with MDSC and tensile tests results. A-6 (acetyl trihexyl citrate) and B-6 (butyryl trihexyl citrate) plasticizers with a weaker softening effect in PHB/PLA matrix have apparently less compact structure with huge holes on the micrographs, especially in the case of A-6 (Figure 10d,e). Larger A-6 and B-6 plasticizers molecules probably are not uniformly miscible with the polymer matrix, therefore they have higher viscosity and such material is predisposed not to fuse well in 3D printed laminas at the same printing conditions. But generally, all of these plasticizers are low-molecular and hence applicable in 3D printing. 3D printing process might be improved in order to avoid the presence of voids. Temperature enhancement during printing should decrease viscosity of melting and cause better fluidity. Furthermore diameter measurements of filament incoming to printer jet and continual flow calibration according to these measurements could help with this problem; and, probably also gradient modification of the height of the layers, where the height of the bottom layers (near to the printing bed) are higher than of those at the top of printed sample.

Arrieta et al. [25] inspected fractured SEM micrographs of PLA/PHB/ATBC (A4) films that showed ductile fracture patterns where the plastic deformations were present.

### 3.6. Determination of the Degree of Disintegration

**Figure 11:**
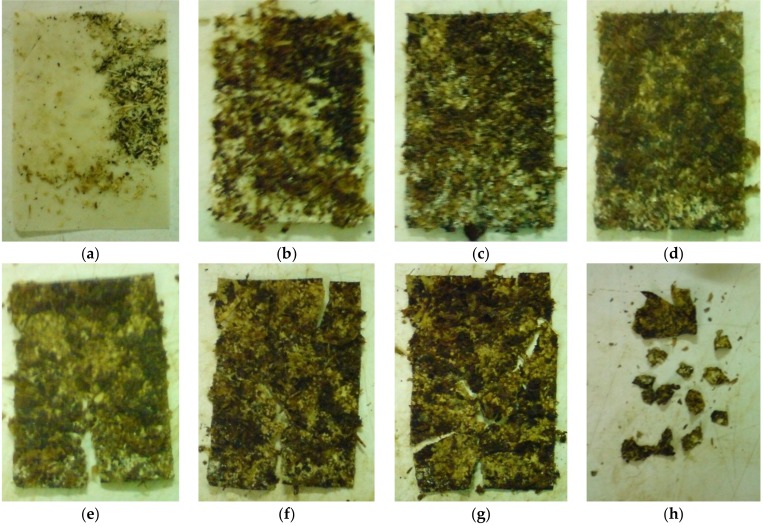
Determination of the degree of disintegration—photos of sample during the test: (**a**) 4th day; (**b**) 12th day; (**c**) 25th day; (**d**) 29th day; (**e**) 32nd day; (**f**) 35th day; (**g**) 42nd day; and, (**h**) 55th day.

This laboratory-scale test was executed according to IS/ISO 20200 under simulated composting thermophilic conditions (at 58 °C). The parameters, like visual appearance, odor, and color were observed and some photos were taken, as can be seen in Figure 11. PHB/PLA/plasticizer sample (60/25/15 in wt %) in the form of film was chosen for this test, with acetyl tributyl citrate as a plasticizer (A-4). The weight of PHB/PLA/A-4 film at the beginning of test was 3.0287 g. Particular sticking of the composting medium on the sample film surface was observed in the 4th day of the experiment. More distinct composting medium sticking on the film surface happened then gradually and was very distinct already in the 12th day of experiment. Concurrently, the medium turned brown. First, small sample degradation signs were observed till the 32nd day of experiment. In the 46th day of experiment, the sample film was smaller only by 5%, but it was very brittle. Subsequently, distinct degradation happened and in the 55th day of the experiment only 25% remained from the original film. Only 5% of the sample remained in the 60th day and the disintegration was completely finished in the 65th day from the test initiation. The subjective rating of the degree of disintegration—preserved sample amount (in %, estimated only by visual observation) can be seen in Table 5.

The samples were disintegrated under thermophilic condition during 65 days. According to IS/ISO 20200, the samples have to be disintegrated until 90 days. Despite positive results, this method is not applicable to the determination of biodegradability of plastic materials under real composting conditions. Still, the determination of the degree of disintegration test brought important information about possible biodegradability under composting conditions.

Other, accurate biodegradable tests of our plasticized matrix with various plasticizers that were performed at water (fresh and salt) and at soil are running at these days. Namely, that is “Determination of the ultimate aerobic biodegradability of plastic materials in an aqueous medium-method by measuring the oxygen demand in a closed respirometer” (ISO 14851:1999), “Water quality—Guidance for determination of biodegradability in the marine environment” (ISO 16221:2001) and “Plastics-Determination of the ultimate aerobic biodegradability in soil by measuring the oxygen demand in a respirometer or the amount of carbon dioxide evolved” (ISO 17556:2003) [44,45,9]. Extensive results from these tests will be part of another publication.

## 4. Conclusions

PHB/PLA/plasticizer melt blends in the weight ratio (wt %) of 60/25/15 were successfully prepared by the corotating twin screw extruder and then by single screw extruder to achieve blended filaments with defined diameters for further 3D printing. Four commercial low molecular plasticizers based on esters of citric acid were used, and all of them were well miscible with PHB/PLA matrix (with PHB majority). Their significant softening effect and structure influence were confirmed by tensile tests and MDSC measurements. Especially, acetyl tributyl citrate (A-4) and tributyl citrate (C-4) plasticizers improved the elongation at break by 308% and 155%, respectively, when compared to non-plasticized PHB/PLA blends. The dogbone samples for tensile tests were designed and successfully printed by 3D printer (FDM technology). Significant plasticizing effect was confirmed also by tensile tests with 3D printed dogbones, although the values of measured elongation at break of dogbones were lower than those for the samples measured in the filament form. Butyryl trihexyl citrate (B-6) and acetyl trihexyl citrate (A-6) plasticizers also had positive softening effect, but considerably smaller as compared to A-4 and C-4 plasticizers. B-6 and A-6 plasticizers improved the elongation at break by 32% and 16%, respectively. MDSC confirmed that the introduction of low molecular plasticizer into PHB/PLA blend caused greater mobility of macromolecular chains. As a consequence, the crystallization and melting temperatures were shifted to lower values. Butyl esters of citric acid caused a decrease of PLA *T*_g_ by 35 °C for C-4 and by 33 °C for A-4. Increased length of alkyl chains linked to citric acid conversely showed worse plasticizing effect and it led to higher PLA *T*_g_. The migration tests—the diffusion of all four plasticizers from PHB/PLA matrix were performed. The plasticizers that softened the best, showed also large diffusion after 15 days of exposition to 110 °C in the drying oven. The influence of plasticizer structure on the diffusion was well noticeable. SEM micrographs of 3D printed dogbones fractured by tensile tests showed more compact areas without large holes in the case of samples with A-4 and C-4 plasticizers. PHB/PLA/A-4 sample in the form of film was disintegrated under thermophilic conditions (according to IS/ISO 20200) during 65 days, still, other biodegradability tests have to be done. The goal was to create a new type of eco-friendly PHB/PLA/plasticizer blends and highly improve the poor mechanical properties of neat PHB/PLA blends (with majority of PHB) by the addition of appropriate plasticizer. All four examined plasticizers are applicable and are suitable also for 3D printing technology, but mainly tributyl citrate and acetyl tributyl citrate are the best for the improvement of ductile properties.

## Figures and Tables

**Table 1:** Commercial and chemical names of used plasticizers, their molecular weights, and labels.

Commercial	Chemical Name	Molecular Weight (g∙mol^−1^)	Plasticizer’s Label
Citroflex^®^ 4	Tributyl Citrate	360.4	C-4
Citroflex^®^ A-4	Acetyl tributyl Citrate	402.5	A-4
Citroflex^®^ A-6	Acetyl trihexyl Citrate	486	A-6
Citroflex^®^ B-6	n-Butyryl tri-n-hexyl Citrate	514	B-6

**Table 2:** MDSC and DSC parameters of PHB/PLA/Plasticizer samples and non-plasticized reference.

Sample	MDSC Scan	First Cooling DSC Scan		Second Heating DSC Scan		
	*T*_g, PLA_ (°C)	*T*_c_ (°C)	*H*_c_ (J/g)	*T*_m_ (°C)	*H*_m_ (J/g)	*X*_c_ (%)
REF	58.4	98	54.6	172	64.0	63
C-4	23.3	80	37.1	163	48.3	55
A-4	25.5	83	39.9	164	51.8	59
B-6	49.8	88	46.0	167	57.4	66
A-6	45.9	82	41.4	168	52.7	60

**Table 3:** Young’s modulus (GPa) of measured PHB/PLA/plasticizer samples and PHB/PLA reference.

Sample	Young’s Modulus (GPa)			
	Filaments—7 Days after Preparation	Filaments—40 Days after Preparation	Filaments—after 3 Days at 110 °C	Dogbones—7 Days after Printing
REF	0.57 ± 0.07	0.45 ± 0.12	0.59 ± 0.06	2.41 ± 0.13
C-4	0.16 ± 0.01	0.17 ± 0.00	0.41 ± 0.08	0.66 ± 0.05
A-4	0.15 ± 0.01	0.17 ± 0.01	0.34 ± 0.03	0.69 ± 0.02
A-6	0.32 ± 0.03	0.32 ± 0.03	0.30 ± 0.02	1.16 ± 0.06
B-6	0.33 ± 0.02	0.30 ± 0.02	0.27 ± 0.05	1.46 ± 0.13

**Table 4:** Tensile strength (MPa) of measured PHB/PLA/plasticizer samples and PHB/PLA reference.

Sample	Tensile Strength (MPa)			
	Filaments—7 Days after Preparation	Filaments—40 Days after Preparation	Filaments—after 3 Days at 110 °C	Dogbones—7 Days after Printing
REF	43.3 ± 1.0	42.9 ± 1.9	44.8 ± 0.5	39.1 ± 1.6
C-4	18.9 ± 0.5	20.4 ± 0.6	30.8 ± 2.6	19.8 ± 0.3
A-4	19.5 ± 0.5	20.2 ± 0.8	26.4 ± 1.6	20.3 ± 0.8
A-6	20.1 ± 0.9	22.5 ± 0.4	22.9 ± 0.4	22.1 ± 1.0
B-6	19.8 ± 0.5	20.7 ± 0.5	20.1 ± 0.6	23.5 ± 1.0

**Table 5:** Subjective rating of the degree of disintegration during test—preserved sample amount (in %).

Day	1	4	6	12	14	15	18	20	25	29	32	35	39	42	46	55	60	65
(%)	100	100	100	100	100	100	100	100	100	100	99	98	97	97	95	25	5	0
